# Analysis of the relationship between personality traits and Internet addiction

**DOI:** 10.1192/j.eurpsy.2023.350

**Published:** 2023-07-19

**Authors:** A. M. Cybulska, K. Rachubińska, D. Schneider-Matyka, S. Grochans, E. Grochans

**Affiliations:** ^1^Department of Nursing; ^2^Department of Clinical Nursing, Pomeranian Medical University in Szczecin, Szczecin, Poland

## Abstract

**Introduction:**

Behavioral addictions are a growing problem in the society. Digital technologies are used by people of different ages, and their number is increasing every year. The precise reasons for the development of addictions remain unknown. In the case of behavioral addictions, especially Internet addiction, the significance of personality traits and behaviors predisposing an individual to addiction, such as depressiveness, anxiety, hostile attitude, aggression, impulsiveness, psychotic behaviors, shyness and self-esteem disorders, are emphasized. Personality predispositions play an essential role in Internet addiction.

**Objectives:**

The aim of this study was a general assessment of the level of Internet addiction with regard to personality traits according to the Big Five model by Costa and McCrae among women from the West Pomeranian Voivodeship.

**Methods:**

The study involved 556 women, whose average age was 34 years and who met the inclusion criteria: female sex, age of 18 years or above, place of residence in the West Pomeranian Voivodship, an informed consent for participation in the study, and completion of the set of questionnaires. The study was conducted using a diagnostic poll method with a questionnaire technique. To carry out the analysis both author’s own and standardized tools were used: the author’s questionnaire covering socio-demographic data, the NEO-Five Factor Inventory which assesses the levels of personality traits, and the Internet Addiction Test used to measure behaviors and characteristics related to compulsive use of the Internet.

**Results:**

Most (70.9%) of the women were average users of the Internet, 25% of respondents were at risk of addiction, and the least numerous group of women (4.1%) was addicted to the Internet. Conscientiousness negatively correlated with Internet addiction (r = -0.22; p < 0.001; R2 = 4.9%). Openness to experience was conducive to Internet addiction (r = 0.15; p < 0.001; R2 = 11.1%). A positive link between neuroticism and Internet addictions was established (r = 0.33; p < 0.001; R2 = 11.1%). No significant relationships between Internet addiction and extraversion (r = 0.03; p = 0.45) as well as agreeableness (r = -0.07; p = 0.10) were demonstrated.

**Conclusions:**

The type of personality of the studied women implicated relationships to Internet addiction. Neuroticism might be a personality trait that particularly predisposes to an increased risk of Internet addiction. Openness to experience was conducive to Internet Addiction. Low levels of conscientiousness more often become addicted to the Internet.

**Disclosure of Interest:**

None Declared

